# Histopathologic Overlap between Fibrosing Mediastinitis and IgG4-Related Disease

**DOI:** 10.1155/2012/207056

**Published:** 2012-05-10

**Authors:** Tobias Peikert, Bijayee Shrestha, Marie Christine Aubry, Thomas V. Colby, Jay H. Ryu, Hiroshi Sekiguchi, Thomas C. Smyrk, Ulrich Specks, Eunhee S. Yi

**Affiliations:** ^1^Division of Pulmonary and Critical Care Medicine, Mayo Clinic, Rochester, MN 55905, USA; ^2^Department of Laboratory Medicine and Pathology, Mayo Clinic, Rochester, MN 55905, USA; ^3^Department of Laboratory Medicine and Pathology, Mayo Clinic, Scottsdale, AZ 85259, USA

## Abstract

Fibrosing mediastinitis (FM) and IgG4-related disease (IgG4-RD) are two fibroinflammatory disorders with potentially overlapping clinical and radiological features. In this paper, we looked for histopathologic features of IgG4-RD and enumerated infiltrating IgG4-positive plasma cells within mediastinal tissue biopsies from FM patients. We identified 15 consecutive FM surgical mediastinal tissue biopsies between 1985 and 2006. All patients satisfied the clinical and radiological diagnostic criteria for FM. All patients had either serological or radiological evidence of prior histoplasmosis or granulomatous disease, respectively. Formalin-fixed paraffin-embedded tissue sections of all patients were stained for H&E, IgG, and IgG4. Three samples met the predefined diagnostic criteria for IgG4-RD. In addition, characteristic histopathologic changes of IgG4-RD in the absence of diagnostic numbers of tissue infiltrating IgG4-positive plasma cells were seen in a number of additional cases (storiform cell-rich fibrosis in 11 cases, lymphoplasmacytic infiltrate in 7 cases, and obliterative phlebitis/arteritis in 2 cases). We conclude that up to one-third of histoplasmosis or granulomatous-disease-associated FM cases demonstrate histopathological features of IgG4-RD spectrum. Whether these changes occur as the host immune response against Histoplasma or represent a manifestation of IgG4-RD remains to be determined. Studies to prospectively identify these cases and evaluate their therapeutic responses to glucocorticoids and/or other immunosuppressive agents such as rituximab are warranted.

## 1. Background

IgG4-related disease (IgG4-RD) is recognized to include a growing number of fibroinflammatory disorders [1–5]. Histopathologic evaluation typically demonstrates distinctive cellular fibrosis organized in an irregular whorled pattern (often referred to as “storiform fibrosis”), obliterative phlebitis/arteritis, and prominent lymphoplasmacytic tissue infiltration [6]. Tissue immunostaining and serum IgG-subclass assessment characteristically reveal large numbers of IgG4 producing plasma cells and elevated serum IgG4 levels, respectively [6].

IgG4-RD was first described in the context of autoimmune pancreatitis presenting with obstructive jaundice due to a space-occupying lesion within the pancreas [7, 8]. Since these initial reports, IgG4-RD has been demonstrated to involve various other organs including the biliary tree (sclerosing cholangitis), salivary (sclerosing sialadenitis), and lacrimal glands (sclerosing dacroadenitis) in isolation or in combination (multisystem involvement) [1–5, 9]. IgG4-RD is typically characterized by clinical and radiographic evidence of an idiopathic metabolically active (e.g., fluorodeoxyglucose-avid) space-occupying lesions within different organs [1–5, 9, 10]. Therapeutically, patients with IgG4-RD typically respond to immunosuppressive therapy with glucocorticoids [11].

Fibrosing mediastinitis (FM), also called sclerosing mediastinitis, is a rare syndrome characterized by an aggressive fibroinflammatory process within the mediastinum [12–15]. Progressive fibrosis caused by the proliferation of invasive fibrous tissue within the mediastinum frequently results in compression and functional compromise of vital mediastinal structures [13–15]. Consequently, FM can lead to substantial disease-related morbidity and perhaps even increased mortality [13, 15].

Although the pathogenesis of FM remains unknown, radiographic, serologic, or histopathologic evidence of prior *Histoplasma capsulatum* infection can often be documented. In endemic areas of North America, the majority of FM cases are thought to represent a rare hypersensitivity reaction to this infection [13–18]. Additional infectious triggers implicated in the pathogenesis of FM include other fungal and mycobacterial organisms associated with granulomatous mediastinitis [13–18]. Finally, there are rare immune-mediated (idiopathic) and drug-induced (e.g., methysergide) cases of FM [13–18]. Interestingly, patients with idiopathic immune-mediated FM frequently have other disease manifestations such as retroperitoneal fibrosis or Riedels thyroiditis, all of which have been associated with the IgG4-RD spectrum [6, 13–18]. Except for selected patients with the idiopathic immune-mediated variant of FM, therapeutic successes using systemic glucocorticoids and other immunosuppressive agents are exceptionally rare [14].

Chest computed tomography in “granulomatous-infection-associated” FM characteristically demonstrates focal, commonly calcified, and most commonly right-sided mediastinal mass lesions. This contrasts the diffuse noncalcified mediastinal infiltration classically seen in idiopathic immune-mediated or drug-induced cases [19, 20]. Given the high diagnostic yield of chest radiological evidence of a focal, calcified mediastinal mass compromising other mediastinal structures, diagnostic tissue biopsies are currently largely reserved to exclude alternative diagnoses such as mediastinal malignancies [14, 20].

Mediastinal lymphadenopathy is one of the most frequent extrapancreatic disease manifestations in patients with IgG4-RD [21, 22]. However, up to date only a single case of FM attributed to IgG4-RD disease has been reported in the medical literature. This Japanese patient had a clinical and radiographic presentation consistent with idiopathic immune-mediated FM but demonstrated histopathological changes typical of IgG4-RD, had an elevated serum IgG4 level, and responded favorably to glucocorticoid therapy [23]. Fibrosis within the mediastinum without compression of mediastinal structures has been reported in the context of patients with other disease manifestations of IgG4-RD [24–26].

We and others have recently demonstrated that mediastinal biopsies from consecutive patients with FM frequently contain large numbers of inflammatory cells including a high number of CD138- (syndecan-1-) positive plasma cells [12, 14]. Consequently, FM is now considered to represent a fibroinflammatory rather than a purely fibrotic disease process. Based on these fibroinflammatory changes associated with the local accumulation of plasma cells, we hypothesized that a subset of FM cases may belong in the IgG4-RD spectrum and demonstrate histopathological and immunological changes consistent with IgG4-RD.

## 2. Patients and Methods

A search of the Mayo Clinic pathology database for a histopathological diagnosis of FM (we used the search terms of *fibrosing mediastinitis, sclerosing mediastinitis,* and *mediastinal fibrosis*) between 1985 and 2006 identified 21 biopsy specimens in the Mayo Clinic tissue registry. The medical records of these cases were reviewed and FM cases were defined clinically by the presence of chest radiological evidence of an infiltrative (crossing tissue planes) mediastinal process associated with the invasion or obstruction of mediastinal structures. Based upon evidence of coexisting malignancies, two patients (one with malignant thymoma and one with desmoplastic mesothelioma) were excluded. The histopathology of the remaining cases was independently reviewed by two of the investigators (TVC and ESY) and a diagnosis of FM was confirmed in 15 patients. Four cases were excluded due to the absence of invasive fibrosis. The clinical and radiographic features and the characterization of adaptive immune response (immunostaining for CD3, CD8, CD20, CD138, and S100) of these cases have been described in detail elsewhere [14].

Immunostaining was performed using a DAKO autostaining system (DAKO, Carpinteria, CA, USA). The monoclonal mouse anti-human IgG4 antibody (clone HP6025, dilution 1 : 100, Zymed, San Francisco, CA, USA) was used to stain formalin-fixed, paraffin-embedded tissue sections. In a similar fashion, IgG staining was performed on consecutive tissue sections using the polyclonal rabbit anti-human IgG antibody (IS512, dilution 1 : 10,000, DAKO, Carpinteria, CA, USA).

The presence and number of IgG- and IgG4-positive cells was evaluated in all 15 FM cases. To enumerate IgG and IgG4-positive plasma cells, the entire slide was scanned at low power for the areas with the highest IgG4-positive plasma cell density. For each case, the three high power fields (hpf) with the highest density were photographed using a magnification of 40x (Nikon Eclipse E400 microscope, field diameter 0.55 mm and Olympus DP70 camera, 0.0645 mm^2^). The corresponding areas were also photographed on the IgG-stained slides. The number of IgG4- and IgG-positive plasma cells was determined in 3 hpf by manually counting positive cells on the photomicrographs and cell numbers were averaged. The number of IgG4-positive cells/hpf was determined, and the fraction of IgG4-positive cells of all IgG-positive plasma cells [%] was calculated for each case.

## 3. Definitions

### 3.1. Clinical Definition of FM

Presence of radiological evidence of an infiltrative, space-occupying mediastinal process with associated pulmonary vascular, airway, superior vena cava (SVC), or esophageal compression. Patients with mediastinal malignancies and/or prior mediastinal radiation therapy were excluded [14, 20].

### 3.2. Histological Case Definition of FM

A histopathological diagnosis of FM required the presence of extensive tissue fibrosis. This fibrous tissue typically infiltrates and obliterates adipose tissue with or without patchy mononuclear immune cell infiltration in the absence of malignancy.

### 3.3. Histoplasma Capsulatum Infection

A *conclusive* diagnosis of infection was assumed in the presence of a positive fungal stain (Grocott methenamine silver (GMS)) or culture of the biopsy tissue specimens and/or serologic titer ≥1 : 32 and/or presence of an M or H band by complement fixation/immunodiffusion. A *suggestive* diagnosis was defined as a serologic titer >1 : 8 and/or radiological features (pulmonary, splenic, and/or hepatic granulomas) suggestive of previous granulomatous infection.

### 3.4. Granulomatous Disease

Patients with radiological features of prior granulomatous disease, histological evidence of granulomatous inflammation, or a localized calcified mass lesion within the mediastinum were classified as previous granulomatous disease [20].

### 3.5. IgG4-RD

IgG4-RD was defined using the following definition: *definite case of IgG4-RD*: at least two of the following three histological features: (1) lymphoplasmacytic infiltrate, (2) storiform-type fibrosis, or (3) obliterative phlebitis/arteritis plus ≥50 IgG4-positive cells/hpf with an IgG4^+^/IgG^+^ ratio ≥40%. *Probable case of IgG4-RD*: one histological feature plus ≥50 IgG4-positive cells/hpf with an IgG4^+^/IgG^+^ ratio ≥40%. *Unlikely case of IgG4-RD*: remaining cases. This definition is based on the consensus recommendations provided by a panel of experts during the International Symposium on IgG4-Related Disease, Boston, MA, October 2011 (http://www2.massgeneral.org/pathology/symposium/IgG4_related_systemic_dis.asp).

The Mayo Clinic Institutional Review Board (IRB) approved the study.

## 4. Results

All 15 patients underwent surgical resection/debulking (7 patients) or surgical biopsy (8 patients) at Mayo Clinic, Rochester, MN, between 1985 and 2006 [14].

Histopathological examination universally demonstrated the proliferation of fibrous tissue with associated infiltration of the surrounding mediastinal fat and soft tissues. Surgical sampling of the adjacent lymph nodes was performed in 8 of 15 cases. In 6 cases, the lymph node samples revealed extensive perinodal fibrosis, extending more than 2 mm beyond the capsule. One case exhibited only mild perinodal sclerosis with fibrous reaction less than 2 mm. In another case, perinodal scarring was absent. In 10 of 15 cases histopathologic evidence of prior granulomatous disease with associated necrosis (with or without calcification) and a surrounding dense fibrotic rim was identified. GMS staining detected characteristic yeast forms for *Histoplasma capsulatum* in 6 of 13 cases. The organisms were typically detected in the necrotic areas of granulomas (Table 1).

**Figure 1:**
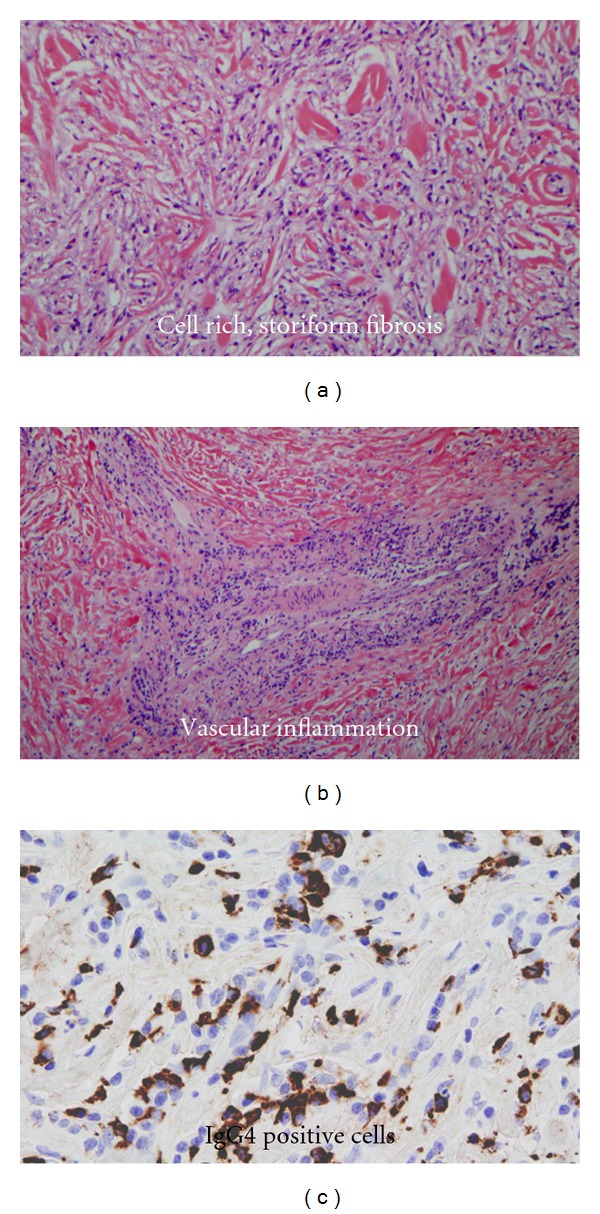
Histopathological changes and IgG4 immunostaining demonstrate an overlap between FM and IgG4-RD in a subset of patients (*n* = 3), (a). cell rich, storiform fibrosis, (b). vascular inflammation and (c). IgG4 positive plasma cells. (representative images selected from 3 patients).

**Figure 2:**
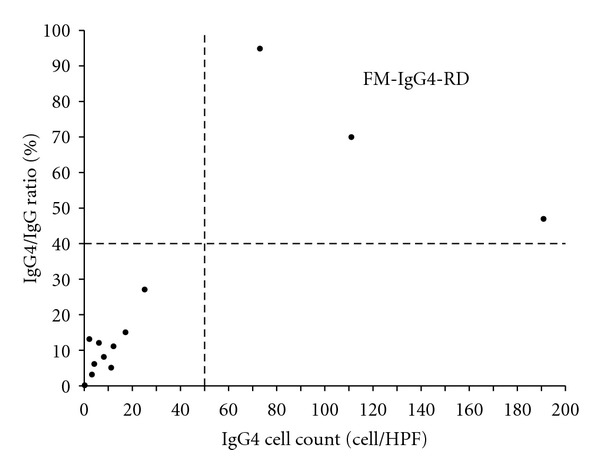
Number of IgG4-positive plasma cells and the corresponding IgG4^+^/IgG^+^ ratios in FM patients (*n* = 15, 3 patients had no IgG4 positive plasma cells).

Three cases met the predefined diagnostic histological criteria for definite IgG4-RD (FM IgG4-RD) (Figure 1). The absolute numbers of IgG4-positive plasma cells and the IgG4^+^/IgG^+^ ratios are summarized in Figure 2. No cases of probable IgG4-RD were identified. Interestingly, despite the absence of the required number and ratio of IgG4-positive plasma cells, characteristic histopathological findings of IgG4-RD such as cell-rich storiform fibrosis and lymphoplasmacytic infiltration were frequently present in the remaining non-IgG4-RD cases (Table 2).

Unfortunately, serum IgG4 levels were not available for any of these patients and none of them was treated with glucocorticoids or other immunosuppressants. The demographic, clinical, and radiological characteristics are summarized in Table 3. Based upon the geographic location of our institution in the Midwestern United States, it is not surprising that all of our cases had evidence of prior granulomatous infections, predominantly histoplasmosis (Table 1). There were no idiopathic immune-mediated cases among our 15 FM patients. Overall there were no significant differences between FM IgG4-RD and FM-non-IgG4-RD cases (Table 3). None of our patients had disease manifestations of IgG4-RD outside the mediastinum.

## 5. Discussion

The precise etiology of FM remains indeterminate. It likely represents a clinical-pathological syndrome attributable to various triggers including infectious pathogens causing granulomatous mediastinitis, drug toxicity and idiopathic immune-mediated cases [13–15]. The majority of FM cases respond suboptimally to antimicrobial, immunosuppressive, and antifibrotic treatments [13–15]. Therapeutic responses of patients with histoplasmosis/granulomatous-disease-associated FM are exceedingly rare. Better understanding of the pathogenesis in specific cases of FM may ultimately result in improved individualized therapies for subgroups of patients.

By definition, IgG4-RD is currently considered an idiopathic fibroinflammatory disorder [1, 2, 4, 6, 9]. It is characterized by the expansion of IgG4-producing plasma cells. The triggers and pathogenesis of IgG4-RD remain undefined [1, 2, 4, 6, 9]. Yet, the disease is typically responsive to glucocorticoid therapy [1, 2, 4, 6, 9]. Furthermore, refractory IgG4-RD cases frequently improve following the depletion of B-lymphocytes with rituximab [11, 27]. Unfortunately, current clinical, serologic, and pathological diagnostic criteria for IgG4-RD, especially outside the pancreas, lack high diagnostic accuracy. The diagnostic criteria for most other organ systems including the mediastinum are exclusively based on expert consensus or extrapolated from observations in the pancreas, salivary, and lacrimal glands. Consequently, a diagnosis of IgG4-RD requires the exclusion of all diseases that can mimic the disorder. Important mimics include malignancies, infections, and vasculitides [28–30].

The clinical, radiological, and pathological presentation of FM as a metabolically active space-occupying fibro-inflammatory disease process within the mediastinum is highly compatible with the disease manifestations of IgG4-RD in other organs such as the pancreas and the salivary glands. Herein we demonstrate that a subset of histoplasmosis/granulomatous-disease-associated FM cases exhibits the histopathological and immunological characteristics consistent with a definite diagnosis of IgG4-RD: lymphoplasmacytic infiltration, storiform fibrosis, obliterative phlebitis/arteritis, and an accumulation of IgG4-positive plasma cells (≥50 cells/hpf and ≥40% of IgG4/IgG-positive plasma cells). Therefore, this subgroup of FM cases may be part of the ever-expanding disease spectrum of IgG4-RD, and perhaps respond favorably to immunosuppressive therapy with glucocorticoids or rituximab. Interestingly, the tissue samples of these patients also contained a large number of CD20-positive B-lymphocytes [14]. Alternatively, these histopathological findings and the expansion of IgG4-positive plasma cells within the tissue biopsies of these patients may represent a subset of an IgG4 dominant fibroinflammatory response triggered by histoplasmosis or other granulomatous diseases. It is conceivable, that this response pattern is indicative of the presence of chronic infection or persistence of foreign antigens within the tissue. Our study has several limitations.

Due to the retrospective design our analysis of the clinical, radiological and serologic data is constrained by the data documented in the medical records by a diverse team of care providers. Furthermore, the infrequent utilization of diagnostic tissue biopsies and surgical debulking procedures in FM patients restricted our analysis of the histopathologic pattern and number of IgG4-positive plasma cells to a relatively small subset of FM patients (small study size). However, we have previously demonstrated that these 15 patients were representative of a large FM cohort treated at a Midwestern tertiary referral center [14]. Our study does not include any tissue specimens from patients with idiopathic immune-mediated FM, which based on the common association with other IgG4-RD and response to immunosuppressive therapy would perhaps be even more likely to represent a manifestation of IgG4-RD. Moreover, serum IgG4 measurements were not performed in these patients, and therapeutic glucocorticoid use was not reported for any of these patients.

In summary, we conclude that there is an overlap of the histopathologic features between histoplasmosis/granulomatous-disease-associated FM and IgG4-RD. A subset, approximately 20%, of these FM cases may indeed be part of the IgG4-RD spectrum. Although the exact pathogenesis and natural history of these cases remains unknown and may differ from classic IgG4-RD, the prospective identification of this subgroup of patients with characteristic IgG4-RD histopathology and a hyper-IgG4 immune response would be extremely valuable. This strategy can perhaps facilitate the identification of a subset of FM patients more likely to respond to immunosuppressive therapy. Consequently, we propose the prospective identification of this subgroup of FM patients based upon the presence of an elevated serum IgG4 level >140 mg/dL and/or characteristic histopathological findings and accumulation of IgG4-positive plasma cells within the mediastinal tissue. As the use of chest computed tomography has largely reduced the need for routine surgical tissue biopsies to establish a diagnosis of FM, based on the vast experience of the successful use of endoscopic needle biopsies for the diagnosis of IgG4-RD in the pancreas, we recommend to obtain transbronchoscopic ultrasound-guided needle biopsies if surgical biopsies are not needed for diagnosis [20, 31, 32]. This approach would allow the identification of a patient population to prospectively evaluate the treatment effects of glucocorticoids and/or other immunosuppressive agents such as rituximab in FM patients with diagnostic features of IgG4-RD (FM IgG4-RD). 

## Figures and Tables

**Table 1:** Radiological, microbiological and histological characteristics of the 15 FM patients.

Age/gender	Histological features of IgG4-RD	Chest radiology	Histoplasmosis/granulomatous disease	Histological granulomatous inflammation
25/M	Definite	Right mediastinal mass	Suggestive	Absent
32/M	Definite	Calcified left hilar mass	Conclusive	Present
65/F	Definite	Right mediastinal mass	Conclusive	Present
51/F	Absent	Calcified left cervical and right hilar mass	Conclusive	Present
31/F	Absent	Calcified right mediastinal mass	Suggestive	Absent
27/F	Absent	Calcified right mediastinal mass	Conclusive	Present
27/F	Absent	Diffuse mediastinal infiltration	Suggestive	Present
48/F	Absent	Left mediastinal mass	Suggestive	Present
35/M	Absent	Calcified bilateral hilar masses	Conclusive	Present
43/F	Absent	Right mediastinal mass	Not available	Present
44/M	Absent	Calcified anterior mediastinal mass	Not available	Absent
27/F	Absent	Calcified right mediastinal mass	Conclusive	Present
59/F	Absent	Calcified right mediastinal mass	Conclusive	Present
36/F	Absent	Calcified right mediastinal mass	Conclusive	Absent
58/F	Absent	Calcified right mediastinal mass	Not available	Absent

**Table 2:** Distribution of the diagnostic criteria of IgG4-RD.

	FM IgG4-RD (*N* = 3)	FM-non-IgG4-RD (*N* = 12)
≥50 IgG4-positive plasma cells/HPF and ≥40% IgG4/IgG ratio, case (%)	3 (100)	0 (0)
Lymphoplasmacytic infiltrate, cases (%)	3 (100)	7 (58)
Storiform, cell-rich fibrosis, cases (%)	3 (100)	11 (91)
Obliterative phlebitis/arteritis, cases (%)	2 (66)	2 (16)

**Table 3:** Demographic, clinical, and radiological features of FM IgG4-RD and FM-non-IgG4-RD cases.

	FM IgG4-RD (*N* = 3)	FM-non-IgG4-RD (*N* = 12)	*P*-value
Age (Median, range (years))	32 (25–65)	39.5 (27–59)	NS
Gender (Women, number of cases (%))	1 (33)	10 (83)	NS
Radiological disease localization			
Right	2	7	NS
Left	0	1	
Bilateral	1	4	

Organ compression			
Superior vena cava	1	3	NS
Pulmonary vascular	1	3	
Airway	1	5	
Esophagus	0	1	

Histoplasmosis (*N* = 13)			
Conclusive	2	7	NS
Suggestive	1	2	
Absent	0	1	
